# A novel atraumatic extraction technique using vestibular socket therapy for immediate implant placement: a randomized controlled clinical trial

**DOI:** 10.1007/s10006-022-01089-4

**Published:** 2022-06-20

**Authors:** Noha Ayman Ghallab, Abdelsalam Elaskary, Hossam Elsabagh, Abrar El Toukhy, Hams Abdelrahman, Gillan El-Kimary

**Affiliations:** 1 Department of Oral Medicine & Periodontology, Faculty of Dentistry, Cairo University, 43 Zahraa Street, Giza, 12311 Dokki Egypt; 2 Faculty of Dentistry, Alexandria University, Alexandria, Egypt; 3 Oral Medicine and Periodontology Department, Faculty of Dentistry, Alexandria University, Alexandria, Egypt; 4 MTI University, Cairo, Egypt; 5 Dental Public Health Department, Faculty of Dentistry, Alexandria University, Alexandria, Egypt; 6 Oral Medicine, Periodontology, Oral Diagnosis and Radiology Department, Faculty of Dentistry, Alexandria University, Alexandria, Egypt

**Keywords:** Atraumatic extraction, Immediate implant, Vestibular socket protocol, Periotome

## Abstract

**Purpose:**

This randomized controlled clinical trial compared soft tissue changes following a novel vestibular atraumatic extraction technique (test group) versus the conventional incisal atraumatic extraction approach (control group) while implementing the vestibular socket therapy for immediate implant placement.

**Methods:**

Thirty patients with hopeless maxillary anterior teeth requiring atraumatic extraction were randomly assigned into two equal groups to receive either test or control. Vertical soft tissue alterations in mm were measured at baseline and 12 months post-restoration using intraoral digital scans at three reference points, distal papilla, mid-facial gingival margin, and mesial papilla, as well as pink esthetic scores (PESs) after 12 months.

**Results:**

Vestibular extraction technique showed significant soft tissue improvement and creeping when compared to incisal extraction (*P* < 0.05). The test group showed soft tissue measurements with a mean (± SD) of 0.26 (± 0.58), 0.39 (± 0.64), and 0.05 (± 0.37) mm for the mesial papilla, mid-facial gingival margin, and distal papilla respectively. While the incisal extraction technique demonstrated gingival recession at the distal papilla, mid-facial gingival margin, and mesial papilla of − 0.37 (± 0.54) mm, − 0.32 (± 0.68) mm, and − 0.39 (± 0.59) mm respectively. The overall PESs after 12 months were 12.67 (± 1.59) in vestibular extraction group, while incisal extraction group was 11.40 (± 1.40), with significant difference between them (*P* = 0.03).

**Conclusion:**

This investigation suggests that both studied techniques were successful in the atraumatic extraction of hopeless severely damaged teeth. The novel vestibular extraction technique might be considered an alternative reliable atraumatic extraction approach compared to the conventional incisal extraction when performing the vestibular socket protocol for immediate implant placement with soft tissue enhancement.

## Introduction

Tooth extraction is associated with physiological alveolar bone loss where the bundle bone loss is evident, followed by dimensional changes in the height and width of the alveolar ridge [1, 2]. The post-extraction loss of alveolar bone compromises the functional and esthetic rehabilitation with removable or fixed prostheses, including dental implants [3]. In addition, thin labial plates of bone are more prone to immediate post-extraction bone loss that might be secondary to pervious chronic inflammation, vertical root fractures, periodontal diseases, and severe trauma before or during extraction [4]. The extent of alveolar bone loss depends on many factors including the following: patient’s general health condition, oral habits; tooth phenotype and location; preoperative condition of the socket; thickness of the buccal bone and post-extraction treatment protocols [3, 5].

It is well established that the mode of extraction influences the extent of alveolar bone resorption [6, 7]. Conventional tooth extraction techniques, involving the use of elevators, luxators, and forceps, all share the concept of socket dimensional expansion. This often leads to fracture or deformity of the interproximal bone with difficulty in maintaining the socket integrity in addition to traumatizing the socket related soft tissues including the interdental papillae, thus, impeding successful implant placement and subsequently challenging future prosthetic replacement. Moreover, extraction of remaining roots or broken teeth with the margin located below the gingival levels can be challenging and emphasizes the priority to preserve the surrounding soft and hard tissues during tooth extraction. In such cases, the standard approach for extraction might involve reflection of a mucoperiosteal flap, often followed by bone removal representing additional alveolar bone loss [8, 9]. Therefore, inducing minimal trauma during hopeless tooth extraction is crucial to preserve the related hard and soft tissue around the tooth, having a significant impact on treatment planning, outcome, and prognosis [10].

Accordingly, various techniques of atraumatic tooth extraction have been introduced in the literature aiming to preserve the bone and gingival architecture, thus allowing appropriate immediate implant placement using a variety of tools like periotomes [9], piezo surgery [11], piezotome [12], and vertical extraction systems [13–16]. Careful atraumatic extraction is also indicated when there is a fracture of the tooth at or below the gingival level and in cases with thin bony plate, this helps keeping minimal changes in soft tissue contour and volume and in turn satisfactory esthetic results [16]. Nevertheless, failure of tooth extraction has been reported in some of these techniques due to retention inadequacy of the screw and root fractures in endodontically treated teeth [9].

Evidence regarding the effectiveness of atraumatic extraction techniques is mainly based on previous case reports or case series [11, 13, 14, 16, 17] while very few studies compared them to conventional tooth extraction [9, 18]. Randomized controlled clinical trials comparing different atraumatic extraction techniques are very scarce, with only one study assessing the clinical efficacy of piezotome versus periotome extractions of non-restorable endodontically treated teeth [12].

Based on the abovementioned data, this investigation proposed a novel procedure for atraumatic tooth extraction, namely vestibular extraction therapy, as it serves a great benefit while implementing vestibular socket protocol for immediate implant placement [19]. The currently proposed technique presents a comprehensive treatment approach for restoring hopeless teeth with severely destructed teeth. To investigate atraumatic extraction, this randomized controlled clinical trial evaluated the vestibular extraction approach versus conventional incisal extraction technique in relation to vertical soft tissue changes after 1 year.

## Material and methods

### Study population

**Fig. 1:**
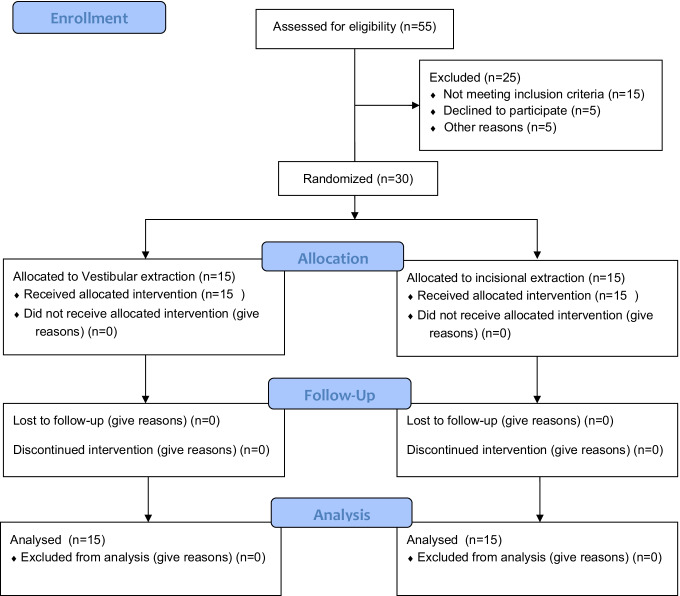
CONSORT flowchart of the study

This randomized clinical trial was registered in Clinical trials.gov (ID: NCT04990999), approved by Central Research Ethics Committee of Supreme Council of University Hospitals, Egypt (No. 0314), conducted in accordance with the Helsinki Declaration of 1975, as revised in 2013 and reported according to CONSORT guidelines, 2012 [20] (Fig. 1). This study included 30 patients (8 males and 22 females, aged 18–57 years) recruited from a private practice clinic in Alexandria, Egypt, between April 2019 to March 2020 meeting the following inclusion criteria: patients having a single non-adjacent hopeless maxillary anterior tooth missing coronal tooth structure, type II socket (deficient labial plate of bone and intact overlying soft tissues), adequate palatal bone, ≥ 3 mm apical bone to engage the immediately placed implants, thereby achieving optimum primary stability (a minimum of 30 Ncm insertion torque) following tooth extraction. Exclusion criteria included the following: smokers, pregnant women, patients with systemic diseases, infected sockets, and history of chemotherapy or radiotherapy within the past 2 years. Eligible patients were informed about the nature of the study and signed a written informed consent to participate in this trial.

### Randomization and blinding

Sequence generation was executed using simple randomization by generating numbers from 1:30 using random allocation software [21] by an investigator (AH) not involved in recruitment nor treatment procedures. Allocation concealment was implemented by the same investigator using sequentially numbered, opaque, sealed envelopes handled to the surgeon (AA) who did not open them until the beginning of interventions. After recruitment, eligible participants who agreed to be included in the study were randomly assigned into two equal parallel groups with a 1:1 allocation ratio to receive either vestibular root extraction (test group) or incisal extraction (control group) based on the generated sequence. Due to the differences in techniques, the operating surgeon (AA) could not be blinded to the procedure. The participants, outcome assessor (TA), and statistician (GN) were blinded.

### Pre-extraction procedure

Prior to tooth extraction, small VOF cone beam computed tomography (CBCT) images (Carestream Health, CS 8100 3D System), with a high contrast resolution detector (high bit depth) and a field of vision 6 × 8, were taken to inspect the overall socket condition. Imaging protocol was standardized by radiographing the patients with a wax interocclusal record to separate maxillary and mandibular teeth at a KVp between 5 and 10. These specifications decreased the beam hardening effect. All patients were scanned pre-operatively using an intra-oral scanner (IOS) (TRIOS, 3Shape A/S, Copenhagen K, Denmark).

### Extraction protocols

Patients assigned to the control group received incisal extraction technique, where atraumatic tooth extraction to the hopeless tooth was performed using periotomes followed by conventional forceps. While in patients assigned to the intervention group, the hopeless tooth was extracted using the novel vestibular root extraction (VRE) technique based on the randomization sequence. In the incisal extraction group, following profound anesthesia administration, a sulcular releasing incision using a micro scalpel (Stoma, Storz am Mark GmbH, Emmingen-Liptingen, Germany) was made to detach the surrounding tissues. A periotome (Stoma, Storz am Mark GmbH, Emmingen-Liptingen, Germany) was inserted interproximally beneath the gingival margin between the bone and the root surface and the periotome blade was maintained parallel to the long axis of the tooth. Then, the periotome was moved horizontally right and left to cut the periodontal ligaments and was then pushed and inserted further apically until sufficient tooth mobility was attained that allowed effortless and seamless completion of the tooth extraction procedure by conventional forceps.

**Fig. 2:**
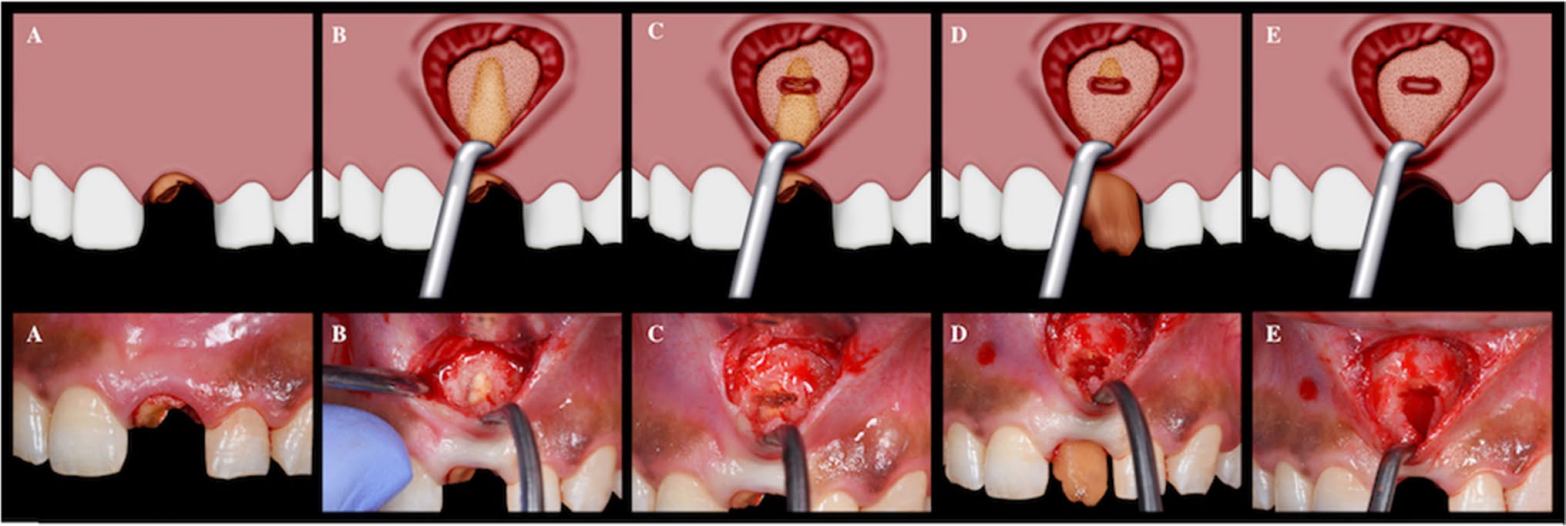
Vestibular extraction technique; **A**–**E**: **A** hopeless tooth to be extracted, **B** vestibular access incision, **C** slit osteotomy at the apical third of the root, **D** pushing the larger segment of the root coronally, **E** removal of the small segment of the root

Regarding the test group, the novel technique of vestibular root extraction was performed (Fig. 2). A 1 cm long vestibular access incision was cut 3–4 mm apical to the mucogingival junction of the hopeless tooth. The vestibular pouch was then dissected in an incisal direction exposing the apical root area and allowing direct undisturbed access to the root surface. Using a long-shanked and small-sized tapered fissure bur, a slit osteotomy was performed at the apical third of the root, separating the coronal two-thirds of the root from the apical one-third. A straight luxator (luxelevator set, Stoma, Storz am Mark GmbH, Emmingen-Liptingen, Germany) was then introduced between the two separated segments, pushing the larger segment coronally, through an axial rotational movement, allowing removal of the root in an incisal direction. The small remaining apical portion was removed using Lucas curette (Stoma, Storz am Mark GmbH, Emmingen-Liptingen, Germany).

### Post-extraction phase

After extracting the hopeless tooth, in both groups, a prefabricated CAD/CAM surgical guide was prepared to deliver the implant (tapered pro Biohorizons, Birmingham, AL, USA) that is known for its aggressive threads design to provide an optimal primary stability, as well as to benefit from its platform switched platform to enhance the peri-implant tissue thickness [22]. An immediate implant (Biohorizons, Birmingham, AL, USA) was installed using a 3D printed surgical guide (Surgical Guide Resin, Form 2, Formlabs). A flexible cortical membrane shield (OsteoBiol® curved Lamina, Tecnoss®, Torino, Italy) of heterologous origin was hydrated, trimmed, and tucked through the vestibular access incision starting at 1 mm beyond the socket orifice and reaching to the apical area of the socket. The gap and/or the defect between the implant body and the shield was then filled with particulate bone graft mix of 75% autogenous bone chips harvested from local surgical sites and 25% inorganic bovine bone mineral matrix (MinerOss X, Biohorizons, Birmingham, AL, USA). The cortical membrane shield was then stabilized to the apical bone using 2 membrane tacks (AutoTac System Kit, Biohorizons Implant Systems, Inc., Birmingham, Alabama, USA). Finally, the vestibular incision was secured with 6/0 nylon sutures (Stoma, Storz am Mark GmbH, Emmingen-Liptingen, Germany). A temporary customized PEEK healing abutment (hexed PolyEtheerEtherKetone Temporary Cylinder, Biohorizons Implant Systems, Inc., Birmingham, Alabama, USA) was trimmed to the socket orifice level and the gap was filled with composite resin (Filtek™ Supreme Ultra Flowable Restorative, 3 M Corporate Headquarters, MN, USA) to seal the bone graft from the oral environment.

### Outcome assessment

**Fig. 3:**
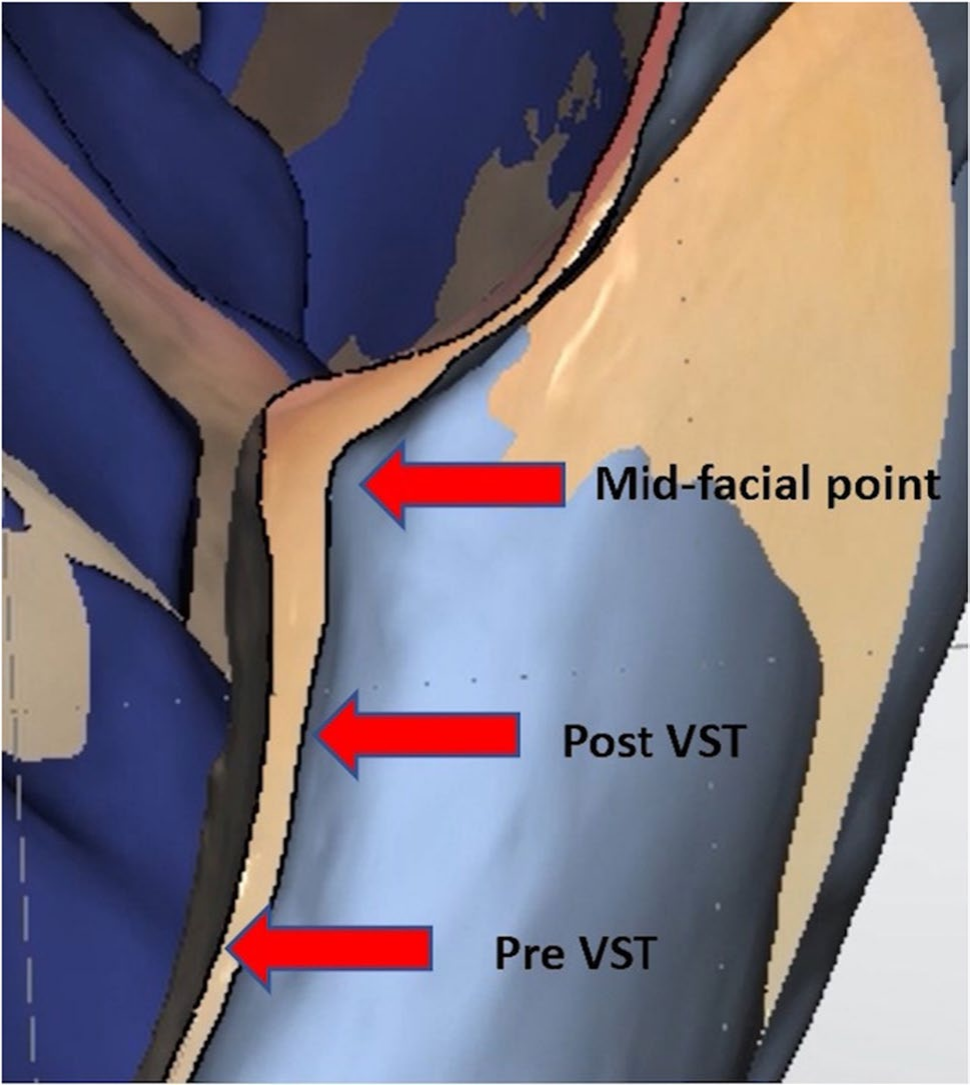
Digital cast measurements superimposing the preoperative and postoperative scans with the aid of a digital software (Madrid, Spain). The red arrow marks of the buccal aspect of the study site on the preoperative and postoperative cast

The main corner stone in this investigation was the soft tissue changes that were evaluated by three measurements, taken at the tip of the mesial papillae, the tip of the distal papillae, and mid-facial gingival margin, on the day of the final restoration delivery and compared to the same measurements taken after 12 months. The changes in the soft tissue height in mm were identified in the 3 reference points by superimposing the baseline file with the postoperative one using the STL (Standard Triangle Language) files of the models obtained via IOS. The 3D software (NemoSmile Design 3D, Nemotec, Madrid, Spain) roughly aligned the baseline and postoperative models through 3 identical points, identified on their surfaces. The best-fit algorithm of the software perfected the superimposition process (Fig. 3). The superimposed models were then imported into an STL viewer (3Shape Ortho viewer, 3Shape, Denmark). The pink esthetic score (PES) [23] was also assessed in this investigation. It was assessed after 12 months by two independent well-trained examiners (EH, EG) with a good intra-examiner agreement (0.82 k value). The PES matches the gingival esthetics around an implant-supported restoration to the contralateral natural tooth. This score comprises seven domains: mesial papilla, distal papilla, soft tissue level, soft tissue contour, deficient alveolar process, soft tissue color, and texture. Each domain is recorded from 0 to 2, with 2 as the best score. The total PES is the sum of the seven domains’ scores, ranging from 0 to 14 (14 is the best score indicating almost similar appearance to the contralateral natural tooth).

### Statistical and power analysis

A total sample size of 24 patients was calculated to detect a mean difference in soft tissue height of 1.2 with SD of 1 based on data from a pilot research, with level of significance *α* = 0.05 and 80% power which was increased to 30 patients to compensate for dropouts (Power and Sample Size program: biostat.mc.vanderbilt.edu/twiki/bin/view/Main/Power Sample Size). Data were explored for normality by Kolmogorov–Smirnov and Shapiro–Wilk tests and presented as mean, standard deviation (SD), mean difference, 95% confidence interval (CI), and frequencies and percentages. Unpaired Student’s *t*-test was used for quantitative data and chi-square test was used for qualitative data. Significance level was set at *P* ≤ 0.05. Statistical analysis was performed with IBM SPSS Statistics (IBM SPSS Statistics for Windows Version 23.0, Armonk, NY: IBM Corp.)

## Results

Patient demographic and clinical data are shown in Table 1. The extraction procedures were performed in 30 patients (22 females and 8 males), with a mean age of 40.23 (± 10.44) years. Table 2 shows vertical changes of soft tissue in mm after 12 months in both studied groups. Regarding vestibular extraction group, the clinical measurements after 12 months revealed soft tissue creeping at the three measured aspects: distal papilla, mid-facial gingival margin, and mesial papilla. The mesial papilla had a mean (± SD) improvement of 0.26 (± 0.58) mm. Similarly, an average improvement of 0.39 (± 0.64) mm was found in the mid-facial gingival margin, while a minimal creeping of the soft tissues of 0.05 (± 0.37) mm was found at the level of the distal papilla. On the contrary, incisal extraction technique showed gingival recession in all measurements. The mm mean (± SD) vertical changes of soft tissue at the distal papilla, mid-facial gingival margin, and mesial papilla were − 0.37 (± 0.54) mm, − 0.32 (± 0.68) mm, and − 0.39 (± 0.59) mm respectively. The current statistical analysis showed that the vestibular extraction technique provided significant post restorative soft tissue marginal stability in the soft tissue dimensions compared to incisal extraction technique at all reference points (*P* < 0.05). Table 3 shows the individual and overall PESs after 12 months. The vestibular extraction group showed PESs of 12.67 (± 1.59), while incisal extraction group scores were 11.40 (± 1.40), with a statistically significant difference detected between them (*P* = 0.03).

**Table 1:** Patient demographic and clinical data

	Vestibular extraction *n* = 15	Incisal extraction *n* = 15	*P*-value
Age (years) Mean (± SD)	37.4 (± 11.44)	43.07 (± 8.83)	0.14
Male *n* (%)	5 (0.33)	3 (0.2)	0.41
Female *n* (%)	10 (0.66)	12 (0.8)	
Central incisor (*n*)	8	7	-
Lateral incisor (*n*)	4	8	
Canine (*n*)	3	-	
Implant survival *n* (%)	15 (100)	15 (100)	1

**Table 2:** Vertical changes (in mm) of soft tissue after 12 months in both studied groups

	Vestibular extraction Mean (± SD)	Incisal extraction Mean (± SD)	Mean Difference [95% CI]	*P*-value
Distal papilla	0.05 (± 0.37)	− 0.37 (± 0.54)	0.42 [0.07, 0.76]	0.019*
Mid-facial gingival margin	0.39 (± 0.64)	− 0.32 (± 0.68)	0.71 [0.21, 1.20]	0.006*
Mesial papilla	0.26 (± 0.58)	** − **0.39 (± 0.59)	0.65 [0.21, 1.08]	0.005*

^*^Significant at *P* ≤ 0.05

**Table 3:** Pink esthetic score (PES) after 12 months in both studied groups

	Vestibular extraction Mean (± SD)	Incisal extraction Mean (± SD)	Mean Difference [95% CI]	*P*-value
Mesial papilla	1.65 (± 0.49)	1.47 (± 0.52)	0.27 [− 0.10, 0.63]	0.15
Distal papilla	1.65 (± 0.59)	1.83 (± 0.39)	0.33 [− 0.13, 0.79]	0.15
Soft tissue level	2.0 (± 0.00)	1.87 (± 0.35)	0.13 [− 0.05, 0.32]	0.15
Soft tissue shape	1.87 (± 0.35)	1.60 (± 0.51)	0.27 [− 0.06, 0.59]	0.11
Deficient alveolar process	1.8 (± 0.41)	1.93 (± 0.26)	− 0.13 [− 0.39, 0.12]	0.29
Soft tissue color	1.87 (± 0.35)	1.73 (± 0.46)	0.13 [− 0.17, 0.44]	0.37
Soft tissue texture	1.80 (± 0.41)	1.53 (± 0.52)	0.27 [− 0.08, 0.62]	0.13
Total PES	**12.67 (± 1.59)**	**11.40 (± 1.4)**	**1.27 [0.15, 2.39]**	0.03*

## Discussion

Immediate placement of dental implants into fresh extraction sockets has proven to be a successful and predictable treatment option in class I sockets with thick buccal plate of bone and related soft tissues using different surgical and loading protocols. It offers superior esthetic and functional advantages as it shortens the treatment duration, reduces the number of surgical visits, preserves soft tissue and hard tissue architecture, and enhances esthetic outcome. Since successful immediate implant placement depends on the buccal bone that remains after tooth/root extraction, this can only be achieved if strict guidelines for atraumatic intervention and preservation of existing anatomic structures are carefully followed [5, 7].

Several factors affect the resorption of alveolar bone crest in immediate implantation including the thickness of the buccal bone wall, the gingival tissue thickness, the degree of periosteal reflection, distance from the implant platform to the crestal bone, surface coating and designs, and the size of gap between the implant and the wall of the alveolar socket [24]. Type II extraction socket presents with intact soft tissue but with dehiscence in labial plate of bone and often results in poor esthetic outcomes with immediate implants being the most difficult to diagnose and it may be mistakenly treated as type I socket [25].

Evaluation of novel surgical techniques is essential to prevent widespread clinical application without being supported by adequate evidence. Nevertheless, evaluation of surgical innovation is challenging since several novel surgical approaches, instruments, and devices continue to develop in our daily clinical practice [18, 26]. In 2020, Elaskary et al. [19] introduced vestibular socket therapy (VST), a novel minimally invasive surgical technique that allowed placement of immediate implant in maxillary class I and class II fresh extraction sockets with or without infective signs, showing predictable esthetic outcomes. The evolution of this technique for immediate implant placement in the esthetic zone was mainly to overcome the remodeling sequelae of tooth extraction and to minimize the mid-facial peri-implant gingival recession [19]. Recently published prospective 2-year follow-up clinical studies provided an evidence for long-term stability of both bone and soft tissue architectures with predictable radiographic, esthetic, and periodontal parameters, advocating VST for immediate implant placement in class I and class II fresh extraction sockets with or without signs of infection [27, 28]. To the best of the authors’ knowledge, this is the first randomized clinical trial comparing the novel vestibular extraction technique to the conventional incisal extraction (using periotomes and forceps) as means of atraumatic extraction of severely destructed teeth implemented along with the vestibular socket protocol for immediate implant placement.

Although several atraumatic techniques for tooth extraction with or without special devices have been introduced in the literature [6, 9, 16], these techniques showed variable inconsistent outcomes where any luxating movement in a horizontal direction or a rotation may result in some socket bone expansion [29]. Other drawbacks were reported as the lengthy procedure of extraction and the operator fatigue [12].

No tooth extraction technique could be completely atraumatic. The only possible exception could be orthodontic extrusion where orthodontic forces result in exfoliation rather than extraction of the tooth [13, 30]. Accordingly, several vertical extraction systems were developed and are currently available in clinical practice including the following: Physics forceps [31], Benex control-root extraction [14], Easy X-TRAC [13], and Sapian root removal device system [15]. These techniques were used in extracting teeth with fractured or damaged crowns that were not suitable for standard forceps extraction procedure. The common principle of these systems is the use of a screw placed in the root of the tooth to be extracted and a mechanism is then applied that allows traction force to be transmitted to this screw along the long axis of the root. In such techniques, there is no direct trauma to the socket walls, as severance of the periodontal ligament is achieved by pulling the conical root in a vertical direction from its socket in a controlled and measured manner without bone expansion [13, 14].

However, vertical extraction systems do hold some clinical limitations. Muska et al. [14] concluded that failure to complete an extraction with the Benex system could be attributed to one of the two main reasons. Firstly, the root morphology may not be compatible with vertical extraction which is often impossible to ascertain from a standard radiograph. Secondly, failure may occur because of insufficient retention of the screw and/or the root fractures. This can either be due to caries or failure to place the screw in an ideal position into the center of the root. Proper case selection, knowledge in using the device, and implementation of that knowledge in treatment planning are important factors in ensuring the success of the Benex system. The relatively higher failure rates observed for maxillary lateral incisors and premolars are also consistent with these explanations [18].

This randomized clinical trial assessed the vestibular extraction technique as a novel approach for atraumatic tooth extraction while applying vestibular socket protocol for immediate implant placement. The present investigation observed that both techniques were successful in the atraumatic tooth extraction of hopeless severely damaged teeth. This is consistent with previous studies reporting successful extraction of badly destructed teeth whether with conventional atraumatic approaches [6, 9, 11, 12] or when vertical extraction systems were used [13–16, 18]. Thus, it might be suggested that this novel concept supports the biomechanical rationale for atraumatic extraction in a similar manner to the vertical extraction systems, yet without the need of the costly tools and devices and the previously mentioned drawbacks.

The current statistical analysis revealed significant post-restorative soft tissue marginal stability in the vestibular extraction technique group compared to the incisal atraumatic tooth extraction group. These observations might be attributed by several factors: mainly the nature of the vestibular extraction approach as it does not cause any trauma, pressure, or laceration to the soft tissue margin, which in turn has a positive impact on enhancing the mid-facial soft tissue levels, as the root was only pushed in an incisal direction through the created vestibular pouch, thus minimizing the osteoclastic activity around the socket rim [7]. Furthermore, the S-shaped prosthetic emergence profile at the peri-implant soft tissue locations leads to an increase in the connective tissue band size that minimizes the likelihood of mid-facial recession around the implant related tissue complex. Moreover, the vestibular extraction technique is a step in the vestibular socket protocol for immediate implant placement [27, 28], which includes grafting the defective labial plate of bone and using a cortical shield that is stabilized securely at the apical bone and reach incisally 1 mm below the gingival zenith. These bone augmentation techniques might also help in stabilizing the gingival tissues and alter the preexisting biological width favorably that is reflected positively to the mid-facial soft tissue levels. On the contrary, the conventional atraumatic extraction technique, using periotomes and forceps from an incisal approach, mainly depends on the degree of the osseous housing expansion, which may result in weakening, or even fracture of the thin labial plate of bone, in addition to, laceration and discrepancy in soft tissue margin [14].

In this study, the magnitude of post-restorative soft tissue recession and esthetic outcomes were evaluated using PES [23]. The currently presented findings demonstrated that the overall PES values after 12 months were 12.67 in the vestibular extraction group, while the conventional incisal extraction group showed scores of 11.4, with a statistically significant difference observed between both studied groups. This suggested that optimum implant esthetics were achieved when the vestibular socket protocol for immediate implant placement was performed. Similarly, Elaskary and coworkers [19, 28] observed satisfying esthetic outcomes with good PES scores (11.33 and 12.63 respectively) after using the vestibular socket therapy for treating intact and compromised fresh extraction sockets with immediate implant placement. A noteworthy outcome in the current clinical trial was the 100% soft tissue level score observed in the vestibular extraction group, with mid-facial soft tissue creeping after 12 months, which was consistent with the previous studies [19, 28]. In agreement with Elaskary et al. [19], the enhanced soft tissue outcome may be explained by the facial bone crest at the implant platform.

The current randomized controlled clinical trial proposes that in situations where vestibular socket protocol for immediate implant placement is applied, both the vestibular extraction and conventional incisal extraction techniques are reliable choices for extracting teeth with severely damaged crowns. Furthermore, the vestibular atraumatic extraction technique improved the surrounding soft tissues, providing stable gingival architecture for subsequent tooth replacement. Despite the plausible results obtained in the present study, there are few limitations that must be addressed. It should be noted that the vestibular extraction technique is not applicable for multirooted teeth. One of the other drawbacks of this investigation was that we could not compare it with any other study, owing to the novelty of the vestibular socket protocol. Furthermore, investigations are also recommended to examine the validity and efficacy of this novel approach in mandibular dense compact bone.

## Conclusions

Within the limitations of the current randomized controlled clinical trial, it might be concluded that vestibular extraction technique could be used as an alternative apically driven atraumatic extraction approach for extracting teeth unsuitable for forceps extraction. Overall, the vestibular extraction protocol for removing subgingivally located hopeless teeth to be restored with immediate implant placement using the vestibular socket therapy approach, enhanced the mid-facial soft tissue levels as wells as PESs compared to immediate implant placement with the conventional incisal extraction approach. Future randomized clinical trials with larger sample sizes and longer follow-up periods are warranted to confirm the findings presented herein and to evaluate the degree of postoperative bone loss following atraumatic tooth extraction.
